# *Bacillus subtilis* spore surface display enhances manganese peroxidase stability and stress resistance

**DOI:** 10.1186/s40643-025-00901-9

**Published:** 2025-06-10

**Authors:** Lu He, Mati Ullah, Muhammad Naeem, Zhong Ni, Yong Feng, Tawaf Ali Shah, Molalign Assefa, Khalid S. Almaary, Huayou Chen

**Affiliations:** 1 School of the Life Sciences, Jiangsu University, Zhenjiang, Jiangsu Province China; 2 College of Agriculture Engineering and Food Science, Shandong University of Technology, Zibo, 255000 China; 3 Southern Agricultural Research Institute, Werabe Agricultural Research Center, P.O. Box. 21, Werabe, Ethiopia; 4 Department of Botany and Microbiology, College of Science, King Saud University, P. O. BOX 2455, Riyadh, 11451 Saudi Arabia

**Keywords:** Manganese peroxidase, Spore surface display, Stress tolerance

## Abstract

Manganese peroxidase (MnP) is in increasing demand due to its ability to degrade straw lignin and facilitate the conversion of agricultural waste into valuable feed resources. However, free MnP faces limitations in industrial applications owing to high operational costs, poor stability, and poor resistance to environmental stressors. In this study, MnP derived from *Irpex lacteus* was displayed on the surface of *Bacillus subtilis* spores using the cotB protein as an anchor molecule. Compared with refolded MnP expressed in *E. coli*, the spore-displayed MnP exhibited superior acid resistance, with an optimal pH of 3.5 (compared to pH 4.0 for the free enzyme). Additionally, the immobilized enzyme CotB-MnP retained 73.9% of its initial activity after incubation at 30℃ for 1 h incubation. Notably, immobilization eliminated the inhibitory effects of K⁺ and Zn²⁺ ions, which instead promoted the activity of the spore-displayed enzyme. The reusable spores maintained 69% of their initial activity after three consecutive cycles, highlighting their potential for industrial straw feed processing.

**Graphical Abstract:**

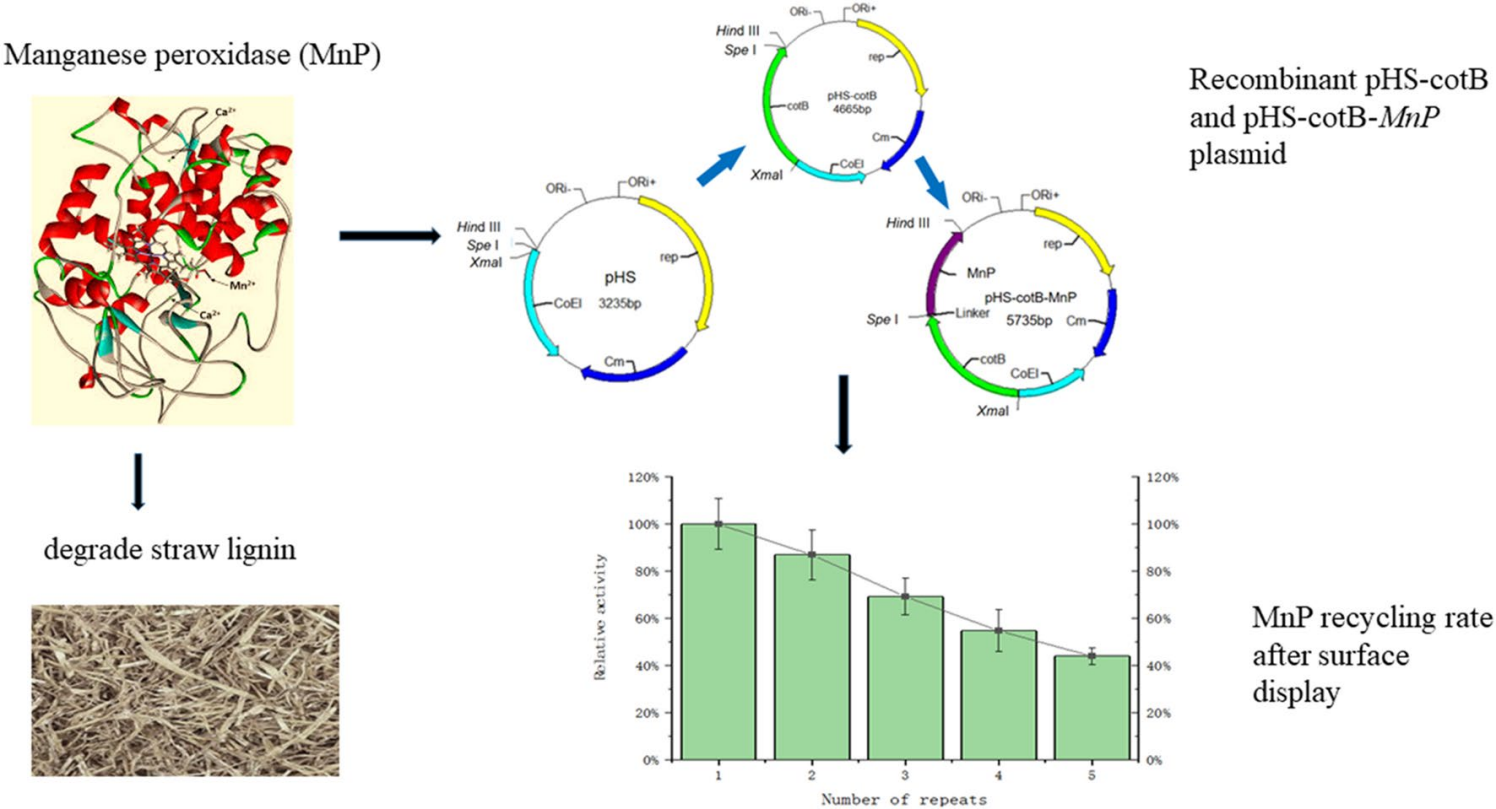

**Supplementary Information:**

The online version contains supplementary material available at 10.1186/s40643-025-00901-9.

## Key points

Enhanced MnP Stability on *B. subtilis* Spores.

Optimal pH Shift to 3.5 for Acid Resistance.

Enhanced Thermostability and Reusability.

**Supplementary Information:**

The online version contains supplementary material available at 10.1186/s40643-025-00901-9.

## Introduction

Lignin is a key structural component of the plant cell wall, forming a complex matrix with cellulose and hemicellulose, collectively known as lignocellulose (Asgher and Iqbal 2013). The chemical stability and intricate structure of lignin have impeded its direct biological utilization, thereby diminishing the nutritional value of agricultural waste such as straw for feed applications (Asgher et al. 2016). Degradation of lignin enhances the accessibility of cellulose and hemicellulose, increasing the nutritional value of straw as a feed resource (Heng et al. 2022). Moreover, organic acids and amino acids produced during lignin degradation are associated with improved digestion and absorption in animals (Øverland and Skrede 2017). It is evident that lignin degradation can improve the digestibility and nutritional value of straw, which is crucial for promoting the use of straw as feed (Li et al. 2024).

Manganese peroxidase (MnP), an extracellular enzyme secreted by white-rot fungi, is essential for the biodegradation of lignin (Wang et al. 2022). MnP catalyzes the oxidation of manganese(II) ions (Mn^2+^) to produce manganese(III) ions (Mn^3+^) (Xiao et al. 2020). These ions form redox-active mediators capable of diffusing to the wood surface, oxidizing phenolic hydroxyl groups, generating phenoxyl radicals, and subsequently forming carbonium ions (Xiao et al. 2020). These ions can further participate in reactions that lead to the cleavage of lignin’s chemical bonds, promoting the solubilization of lignin (He et al. 2024). Beyond lignin degradation, MnP also exhibits potential in environmental remediation (Bilal et al. 2017), yet its industrial application remains limited by stability issues.

Spores are dormant cell structures formed by certain bacteria in response to adverse environmental conditions such as nutrient deprivation, extreme temperatures, pH fluctuations, ultraviolet irradiation, and exposure to toxic chemicals (Dworkin and Shah 2010). The mature spore structure, from the inside out, consists of the core, cortex, spore coat, and exosporium (Guoyan et al. 2019). The core contains bacterial genomic DNA, 2,6-pyridinedicarboxylic acid (DPA), and small, alpha/beta-type acid-soluble spore proteins (SASPs). DPA replaces water in the core and, in conjunction with SASPs, binds to DNA, significantly enhancing the spore’s thermotolerance and ultraviolet resistance, and maintaining the dehydrated state (Lin et al. 2020), dormancy, and revival of the spore. The cortex is primarily composed of specially modified peptidoglycan, while the spore coat, composed of approximately 70 proteins, endows the spore with resistance to chemical reagents. Despite their extremely low metabolism, spores can sense environmental changes and, under favorable conditions, germinate to form new vegetative cells (Isticato and Ricca 2014). This unique structure and function enable spores to survive and germinate under stress, providing a crucial mechanism for bacterial survival and reproduction in extreme environments (Wu et al. 2024).

*Bacillus subtilis*, a Gram-positive, aerobic spore-forming bacterium, is renowned for its robust secretion capabilities, simple cell wall structure, and non-toxic nature. Classified as a non-pathogenic and non-invasive strain, it has been recognized by the U.S. Food and Drug Administration as Generally Recognized As Safe (GRAS). Consequently, *B. subtilis* is extensively utilized as a probiotic and food additive in the food industry and beyond. As a model organism for studying spore-forming bacteria, its spore structure and function have been extensively studied. Bio-immobilized enzymes displayed on the surface of *B. subtilis* spores have garnered significant attention for their potential to improve enzyme stability, reusability, and process simplification. Research indicates that reducing pH during straw fermentation helps suppress methane production and enhance feed digestibility. However, the application of spore-displayed MnP in lignin degradation remains unexplored, particularly in addressing ion inhibition and operational stability.

In the *Bacillus subtilis* spore surface display (BSSD) technology, various coat proteins have been reported for use as anchor proteins for the display of exogenous proteins, including CotB, CotC, CotG, CotZ, CotX, CotY, etc. (Iwanicki et al. 2014). Among these, CotB, CotC, and CotG have been more extensively studied. CotB and CotC are primarily used for antigen display, while CotG is mainly used for the display of active enzyme proteins(Mingmongkolchai and Panbangred 2019). These anchor proteins must meet specific criteria: they should possess domains that ensure the successful display of exogenous proteins on the spore surface, have anchoring domains that can be fused with exogenous proteins to firmly display them on the spore surface; the fusion proteins should not affect the structure and function of the respective coat and exogenous proteins; moreover, they should be capable of resisting proteolysis in the periplasmic space(Isticato et al. 2001). These characteristics make these coat proteins an ideal choice for the display of exogenous proteins in BSSD technology. Leveraging the stress resistance of spores, the displayed exogenous proteins exhibit similar traits, demonstrating higher stability, ease of purification, no need for expression, the ability to express macromolecules or a greater variety of proteins, and a lack of significant codon bias(Kim and Schumann 2009).

In this study, we expressed MnP from *Irpex lacteus* in fusion with the *B. subtilis* spore anchoring protein CotB. CotB was selected as the anchoring protein due to its high surface exposure efficiency and compatibility with large enzymes. The expression and activity, enzymatic properties, and recovery of the enzyme on the surface of recombinant spores were evaluated using various methods to further explore the differences between MnP immobilized by *B. subtilis* spores and the free enzyme expressed by *E. coli*, aiding in the advancement of MnP applications in the field of straw pretreatment and other areas.

## Methods

### Strains, vectors, chemical reagents

Our laboratory maintains the plasmid pHS, a transferable expression vector suitable for both *Escherichia coli* and *Bacillus subtilis*. The *E. coli* strains BL21 and DH5α were sourced from Shanghai Sangon Biotech Co., Ltd. The *Bacillus subtilis* strain WB800N used in this study was maintained for this experiment and showed kanamycin resistance (Kan+). For enzymatic reactions, DNA polymerase was procured from New England BioLabs, USA. T-4 DNA ligase, and the restriction enzymes *Spe* I and *Hin*d III were obtained from TaKaRa, Japan. The specific substrate 2,2’-Azino-bis(3-ethylbenzothiazoline-6-sulfonic acid) (ABTS) was supplied by Sigma Corporation, USA. LB medium was composed of yeast extract 0.5% w/v, tryptone 1% w/v, NaCl 0.5% w/v. The components of DSM medium includes: nutrient broth 0.8% w/v, MnCl_2_ 10 µM, KCl 0.1% w/v, FeSO_4_ 1.0 µM, MgSO_4_·7H_2_O 0.025% w/v, and Ca(NO_3_)_2_·4H_2_O 1.0 mM. All other chemical reagents used were of analytical grade purity. Microbial cultures were performed on LB agar plates supplemented with appropriate antibiotics at 37 °C. Sporulation of *Bacillus subtilis* WB800N was induced in DSM medium at 37 °C with continuous shaking at 220 rpm for 36 h.

### Construction of MnP plasmid in *E. coli*

In this study, the *mnp* gene sequence (GenBank accession no. KC811382.2) was obtained from the NCBI database and optimized to remove its original 31 amino acids, ensuring the mature expression of the *mnp* fragment. Subsequently, the optimized *mnp* fragment was inserted plasmid pET-21a, constructing the recombinant plasmid pET-21a-*mnp*, where the C-terminus of *mnp* was fused with a 6*His tag to facilitate protein purification. Next, the plasmid was transformed into *E. coli* DH5α for initial verification. After successful verification, the plasmid was extracted from DH5α and transformed into *E. coli* BL21(DE3).

### Expression of MnP in *E. coli*

In the experiment, 1 mM of isopropyl β-D-1-thiogalactopyranoside (IPTG) was first used to induce recombinant *E. coli* BL21-pET-21a-*mnp* to promote the expression of MnP protein. After 12 h of induction at 37℃, the cells were separated by centrifugation at 10,000×g for 15 min, and then washed twice with phosphate buffered saline (PBS) to remove media residues and loose cell debris. The bacterial pellet was then resuspended using lysis buffer (100mM NaCl, 50mM Tris-HCl, 0.1mM Dithiothreitol, 5%Glycerol, pH8.0), and the cells were disrupted by sonication at 25% power for 30 min until a clear state was achieved to release the target protein MnP. The inclusion bodies were washed twice with inclusion body wash buffer to remove other cellular components, and the residue was the MnP inclusion bodies.

### Denaturation of MnP inclusion bodies

The washed inclusion bodies were stirred in a refolding buffer (50 mM Tris-HCl, 0.5 mM EDTA, 1 mM NaCl, 10% glycerol) containing 8 M urea for 2 h to denature the MnP inclusion bodies. The solution was then subjected to a stepwise dialysis against a descending scale of urea concentrations, starting from 6 M and sequentially reducing to 3 M, 1 M, 0.5 M, 0.25 M, and finally concluding with 0.1 M. This method was employed to gradually facilitate the refolding and restoration of activity of the target protein, MnP. During the critical refolding stages at urea concentrations of 3 M and 1 M, the system was supplemented with 0.3 mM oxidized glutathione and 1.5 mM reduced glutathione, respectively. After the completion of the refolding process, 10 µM hemin chloride and 25 mM calcium chloride were added to the solution, followed by refrigeration at 4 °C for 10 h to stabilize the protein structure and restore its activity. Finally, the mixture was centrifuged at 10,000×g for 20 min at 4 °C to remove excess hemin chloride and calcium ions.

### Preparation of MnP polyclonal antibodies

This experiment removed low molecular weight impurities such as imidazole using Amicon-Ultra-15 ultrafiltration tubes (Millipore, 10 kDa molecular weight cutoff). The concentrated refolded-MnP protein was then washed three times with normal saline. 300 mg of refolded-MnP protein was mixed with an equal volume of Freund’s complete adjuvant and injected into New Zealand white rabbits via multipoint injection to stimulate their initial immune response. Two weeks later, booster immunizations were administered every two weeks with 100 mg of MnP protein mixed with Freund’s incomplete adjuvant. The booster immunizations continued until the production of adequate titers of anti-MnP antibodies in the rabbits was confirmed by ELISA or other immunological detection methods. In the study, three female rabbits, each with an average weight of around 2.5 kg, were selected for the investigation. These animals were housed in a controlled laboratory environment with unrestricted access to water and food that met standard nutritional requirements. Upon the study’s conclusion, the rabbits were humanely euthanized through exposure to carbon dioxide, a method recognized for its rapid and painless effect. The experimental protocols were thoroughly reviewed and granted approval by the Jiangsu University Institutional Animal Care and Use Committee, with the specific reference number UJS-IACUC-2,023,102,301, ensuring that all procedures adhered to ethical and humane standards.

### Construction of shuttle plasmid and Recombinant WB800N strain

**Fig. 1:**
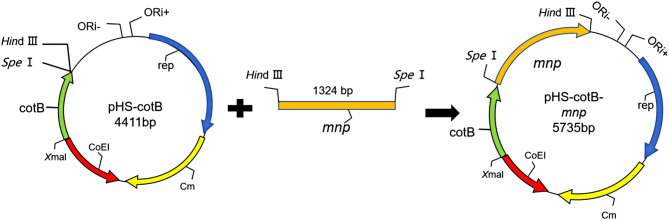
Steps in construction of recombinant pHS-cotB and pHS-cotB-*mnp* plasmid

Genomic DNA of *B. subtilis* 168 was extracted using a Vazyme genomic DNA extraction kit. The cotB fragment was amplified with primers cotB-F and cotB-R Table 1. A flexible linker peptide composed of GGGGS (indicated in italics within the primers) was inserted at the C-terminus of CotB. The *cotB* gene was inserted into the pHS plasmid using the *Xma*I and *Spe*I restriction sites, resulting in the construction of the pHS-cotB plasmid, with the plasmid map depicted in Fig. 1.

**Table 1:** List of primers used in this study

Primer Name	Primer sequence	reference
cotB-F	TAG*CCCGGG*ACGGATTAGGCCGTTTGTCC	This study
cotB-R	GG*ACTAGTTGAACCCCCACCTCC*AAATTTACGTTTCCAGTGATAGTCTATCG	This study
*mnp*-F	GG*ACTAGT*ATGGCCTTCAAACACCTCATCGC	This study
*mnp*-R	CCC*AAGCTT*GCTTCCTTTCGGGCTTTGTTAGC	This study

Based on the *E. coli-B. subtilis* shuttle plasmid pHS containing the coat protein *cotB* gene stored in our laboratory, the synthesized *mnp* gene was used as a template to amplify the *mnp* target fragment (1024 bp, Figure S2,) by PCR using a high-fidelity polymerase. The linearized plasmid (Figure S3,) and the double-digested *mnp* target fragment were then ligated using T4 Ligase to construct the pHS-cotB-*mnp* plasmid, which was subsequently transformed into *E. coli* DH5α.

Take the stored *Bacillus subtilis* WB800N competent cells from the laboratory and place them in a 45 °C water bath for 10 min to allow them to thaw. Withdraw 10 µL of the constructed pHS-cotB*-mnp* plasmid and add it to the competent cells, gently mixing. Set up a control without adding the pHS-cotB plasmid. Mark each competent cell and place it in a 37 °C water bath for 30 min. After half an hour, remove and place in a 37 °C incubator with 170 rpm shaking for 2–3 h. After the culture is finished, centrifuge at 6000×g for 8 min to precipitate the bacterial cells. Retain 100 µL of the supernatant to resuspend the bacterial cells and spread them evenly onto solid agar plates containing chloramphenicol resistance, then incubate overnight at 37 °C in an inverted position. The next day, pick the transformants for testing. After sequencing and confirmation of accuracy, save the bacterial strains for future use.

### Preparation of Recombinant MnP spores

Two recombinant *Bacillus subtilis* WB800N strains (pHS-cotB, pHS-cotB*-mnp*) were cultured in LB medium containing chloramphenicol and kanamycin at 37 °C for 12 h to activate the strains. Subsequently, in 1000 ml of medium, we added 100 ml of bacterial suspension, 1% of the original culture, as an inoculum. A 1% bacterial inoculum was transferred to DSM liquid medium and incubated for 36 h at 37 ℃ and 220 rpm to promote sporulation.

Upon completion of the bacterial culture, the microorganisms were harvested via centrifugation at a speed of 6000×g for 15 min. The resulting bacterial pellet was reintroduced into a GTE buffer solution composed of 50 mM glucose, 20 mM Tris-HCl at a pH of 7.5, 10 mM EDTA, and 2 mg/mL of lysozyme. This mixture was incubated at 37 °C for 45 min to break down the cell wall and liberate the primary spores. Following this, the spores were subjected to a second centrifugation step at 6000×g for another 15 min to separate them from the cell debris. The spores were subsequently washed twice to remove residual contaminants using a suitable washing medium.PBS buffer to remove excess cell wall debris. After the final centrifugation, the spores were resuspended using PBS and stored temporarily at -20 °C.

### MnP enzyme activity assay

MnP activity was assayed in a 50 mM lactate-sodium lactate buffer solution containing 1 mM ABTS, 1 mM MnSO_4_, and 0.1 mM H_2_O_2_. Enzyme activity was measured for both enzymes under standard MnP assay conditions (pH 4.5, 25℃). The enzyme activity was determined by monitoring the oxidation of 1 mM ABTS (ε420 = 36000 L×mol^− 1^×cm^− 1^) in the lactate-sodium lactate buffer by MnP. The reaction was carried out for 10 min, and the absorbance was measured at 420 nm. One unit (1 U) of MnP activity is defined as the amount of enzyme that produces 1 µmol of oxidized product per minute under standard conditions.

To determine the activity of the free complex MnP in *E. coli*, the protein concentration (mg/mL) of the supernatant was measured using the Bradford method to calculate the specific activity of the complex MnP per milligram of protein. A suspension of the free enzyme that was inactivated at 100 °C for 10 min was used as a negative control. Additionally, a reaction mixture without the substrate served as a blank control.

The enzyme activity of the recombinant spore suspension was determined by adjusting the OD_600_ of the spore-containing suspension to 0.8 as a standard for spore measurement. Recombinant spores transformed with vectors lacking the target gene were used as a negative control. Absorbance values were corrected by subtracting the background from control reactions containing heat-inactivated enzyme or no substrate. All experiments were performed in triplicate, and data are presented as mean ± standard deviation. Significant differences (*p* < 0.05) were determined by Student’s t-test using GraphPad Prism 9.0.

### Western blot analysis

To detect the display of MnP protein on the spore surface, the spores were resuspended in a protein extraction buffer containing 1% SDS and 50 mM dithiothreitol. The suspension was then heat-treated at 70 °C for 45 min to extract the proteins exposed on the spore surface. Western blot analysis was performed using a polyclonal antibody against MnP, which was prepared in a previous experiment, as the primary antibody, and horseradish peroxidase-labeled goat anti-rabbit IgG (purchased from Sangon Biotech) as the secondary antibody. The control group consisted of recombinant spores transformed with an empty plasmid treated under the same conditions. Omni-ECL™ alkaline chemiluminescent kit (Epizyme Biotech) was used for color development to observe the expression of MnP protein.

### Immunofluorescence analysis of Recombinant MnP spores

After soaking the glass slides in a 0.1% gelatin solution for 2 min and drying them, the spore suspension was appropriately diluted, and 10 µL was dropped onto the slides. The spores were air-dried to ensure their adhesion to the pretreated slides. Subsequently, the slides were washed three times with PBS to remove unfixed spores. Next, 5% (w/v) non-fat milk powder solution was used to block nonspecific binding. After washing and drying, the slides were incubated with MnP polyclonal rabbit antibodies at 4 °C to specifically recognize the target protein. To detect the presentation of MnP protein on the spore surface, spores were resuspended in protein extraction buffer containing 1% SDS and 50 mM dithiothreitol.

After incubation, the slides were washed twice using PBS and then dried. They were then incubated with fluorescently labeled secondary antibodies at room temperature for 1 h. After incubation, the slides were washed twice more and then dried to visualize the fluorescence of the spores under a fluorescence microscope. Controls were spores obtained from WB800N pHS-cotB.

### Analysis of enzymatic properties of MnP

Under standard enzyme activity assay conditions (pH 4.5, 25℃), we first measured and compared the activities of cotB-MnP and refolded-MnP. To further analyze performance differences between the two enzymes under different states, experiments were sequentially conducted for key reaction parameter screening, stability evaluation, and environmental factor impact analysis. In the investigation of optimal reaction conditions, enzyme activities were tested across a pH range of 2.5–6.0 at 25℃, determining that the optimal pH values for cotB-MnP and refolded-MnP were 3.5 and 4.0, respectively; subsequently, under pH 4.5 conditions (consistent with standard assay conditions), optimal reaction temperatures were identified by measuring activity across a temperature range of 25–55℃. For stability analysis, to examine the effect of temperature on enzyme activity, both enzymes were incubated at a unified 30℃ (based on prior optimal temperature determination results) for 3 h, with activity measured every 30 min and analyzed for time-dependent changes using the initial enzyme activity under standard conditions (defined as 100% activity) as the baseline; for pH stability, identical incubation and measurement procedures were performed at each enzyme’s optimal pH (3.5 for cotB-MnP, 4.0 for refolded-MnP) at 30℃, with stability assessed similarly using initial activity as the baseline. In environmental factor impact experiments, the effects of metal ions, surfactants, and acidic phenolic compounds on enzyme activity were systematically studied under standard assay conditions (pH 4.5, 25℃) to explore protein stability in diverse reaction environments. To ensure comparability and accuracy of experimental data, spore suspensions were standardized to OD_600_ = 0.8 (with a deviation of ± 0.02) to establish a uniform basis for enzyme activity evaluation; enzyme activity was expressed as units per milliliter (U/mL), with each sample tested at least three times and the entire experimental procedure repeated more than three times. Statistical analysis utilized one-way ANOVA combined with Tukey’s post-hoc test (*p* < 0.05) to assess significant differences, ensuring reliable results.

### Recovery and reusability determination of spore-displayed MnP-displaying spores

To evaluate the reusability of the CotB-MnP fusion protein, a series of five consecutive enzymatic activity tests were conducted at 30℃ and pH 3.5. Reusability was assessed using 1 mL of spore suspension (OD_600_ = 0.8) in five sequential reactions under these conditions. After each reaction, spores were harvested by centrifugation at 4500×g for 8 min, washed three times with lactate-sodium lactate buffer (50 mM, pH 3.5) to remove residual ABTS and Mn^2+^, and resuspended in fresh buffer. The activity measured in the first cycle was set as 100% to calculate the relative catalytic efficiency of the immobilized spores. Each sample was analyzed with at least three technical replicates, and the entire procedure was independently repeated three times to strengthen the study’s findings.

## Results

### MnP expression in *E. coli* Recombinant

**Fig. 2:**
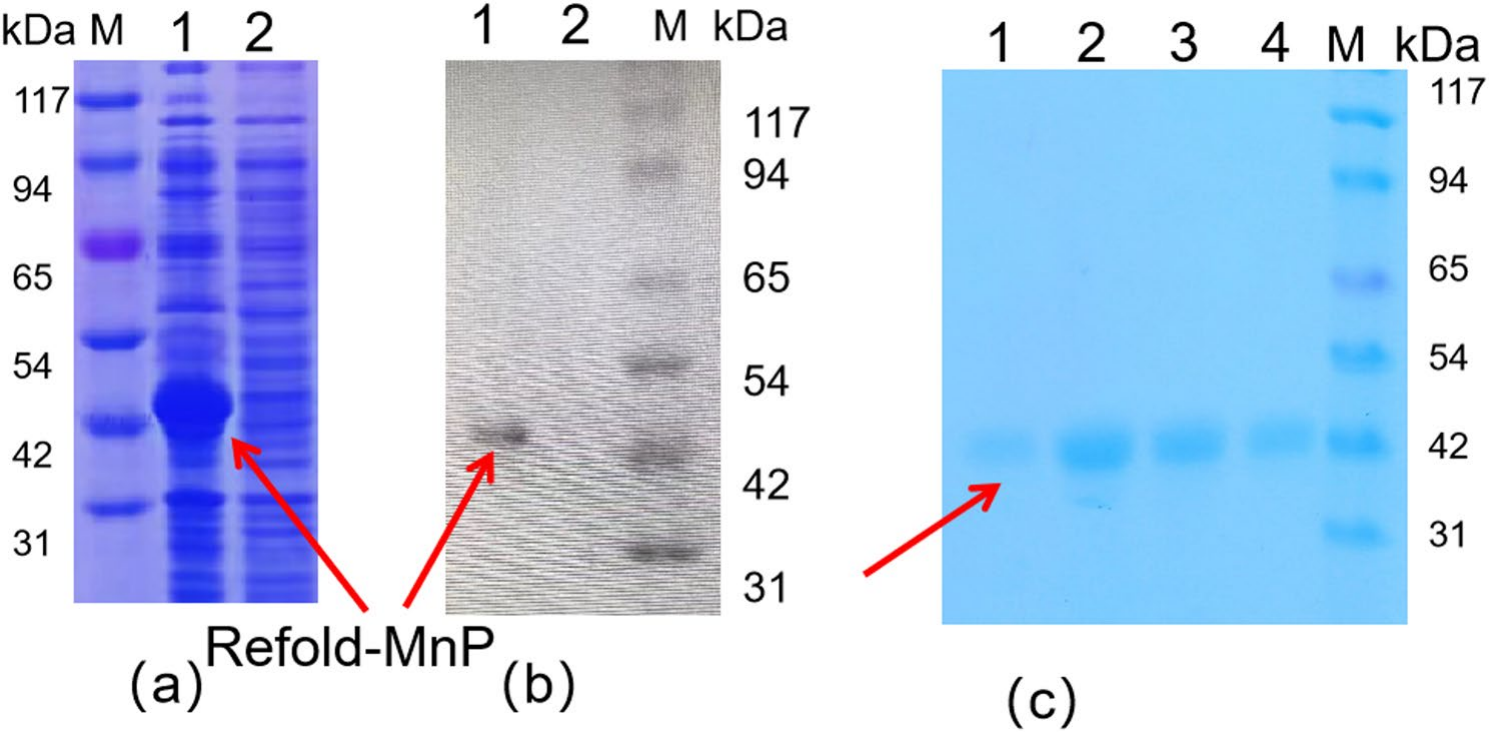
Analysis of MnP Protein Expression and Purification in *E. coli.* SDS-PAGE (**a**) and Western Blot (**b**): Lane M indicates the protein marker from Sangon Biotech. Lane 1 corresponds to the expressed pET-21a-*mnp* precipitated protein; Lane 2 to the pET21a control precipitated protein. Purification (**c**): Lane 1–4 respectively show the purified MnP (43 kDa) eluted by imidazole at 100 mm, 200 mm,300 mm and 500 mm. M: Protein labeling

In this study, we successfully expressed the reorganized MnP protein in the host of *E. coli* BL21 (DE3). The protein carried a 6*His tag to facilitate subsequent purification and detection. As shown in Fig. 2(a), the results of the sodium dodecyl sulfate polyacrylamide gel electrophoresis (SDS-PAGE) analysis confirmed that after IPTG induction, the recombinant *E. coli* BL21-pET-21a-*mnp* efficiently expressed the expected MnP protein with a molecular weight of 43 kDa. This finding is consistent with the results of Zhang (Zhang et al. 2018), who reported successful expression of recombinant protein in the *E. coli* expression system.

Further Western blot analysis using specific antibodies for the 6His tag confirmed the target protein band at 43 kDa, as shown in Fig. 2(b). This result matches the expected MnP protein molecular weight, verifying successful expression and target protein identity. The MnP protein was purified from inclusion bodies using the Ni-NTA affinity chromatography method. Following Ni-NTA affinity chromatography, the target protein was eluted from100mM to 500 mM imidazole, yielding a single band at 43 kDa (Fig. 2(c)). This purification strategy is similar to the method described by Shouzhi Li (Li et al. 2024), who effectively purified other 6*His tag fusion proteins using this method.

The purified MnP protein was further concentrated using ultrafiltration technology. In previous literature, the activity of MnP enzyme obtained by dilution renaturation method after expression in *E. coli* was 0.530 U/mL, while the activity of the gradient renaturation protease used in our study was 53.88 U/mL, significantly improving the efficiency of protein renaturation. Quantitative analysis of the protein showed a concentration of 1.42 mg/mL, as shown in Figure S1. This concentration level is higher than that reported by Muhammad Asghe(Asgher and Iqbal 2013), in similar studies, indicating that our purification process has a high yield. Additionally, we determined the activity of refolded-MnP protein and found it to be 37.94 U/mg. This data is recorded in Table 2. The activity value is higher than that of most MnP proteins reported in previous documents (Moon and Song 2012). For example, the enzyme activity of MnP obtained by Werntingl Chen through homologous expression was 8.0 mU/mg (Chen et al. 2015), while the enzyme activity of MnP obtained by Qin, Xing through heterologous expression and purification in *E. coli* was 24.9 U/mg (Qin et al. 2014), suggesting that our expression and purification strategy may enhance the catalytic efficiency of the protein.

**Table 2:** Enzyme activity of MnP

Enzyme	Enzyme Activity (U/mL)	Protein Content (mg/mL)	Specific Enzyme Activity (U/mg)
Refolded-MnP	53.88	1.42	37.94
CotB-MnP	62.74	ND	ND

To further investigate the function and application of the MnP protein, we used this purified and highly concentrated MnP protein to prepare polyclonal antibodies in New Zealand white rabbits. These antibodies will be used for subsequent immunological research, including localization analysis of MnP protein under different conditions and its potential assessment in biocatalyst and bioengineering applications.

### Characterization of MnP on the spore surface

The plasmids pHS-cotB and pHS-cotB-*mnp*, extracted from *E. coli* DH5α, were transferred into the competent cells of *Bacillus subtilis* WB800N. After the growth of the strains, individual colonies were selected and verified through PCR amplification and gel electrophoresis to ensure the successful integration of the target recombinant plasmids. The verified recombinant spores were then cultivated and induced to produce the desired proteins.

**Fig. 3:**
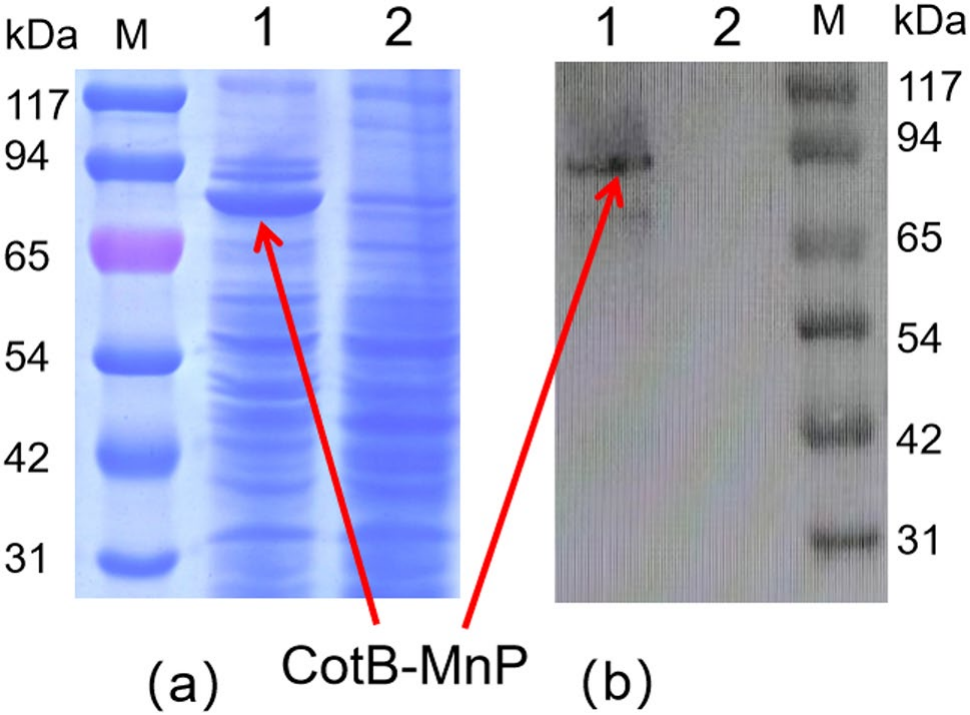
Expression of MnP protein displayed on the surface of *Bacillus subtilis* spores through the CotB anchor protein. SDS-PAGE (**a**) and Western Blot (**b**): M: Protein marker (Shanghai Sangong Biotechnology Co., Ltd.); Lane 1 corresponds to the expressed CotB-MnP fusion protein; Lane 2 is the CotB protein, serving as a control

**Fig. 4:**
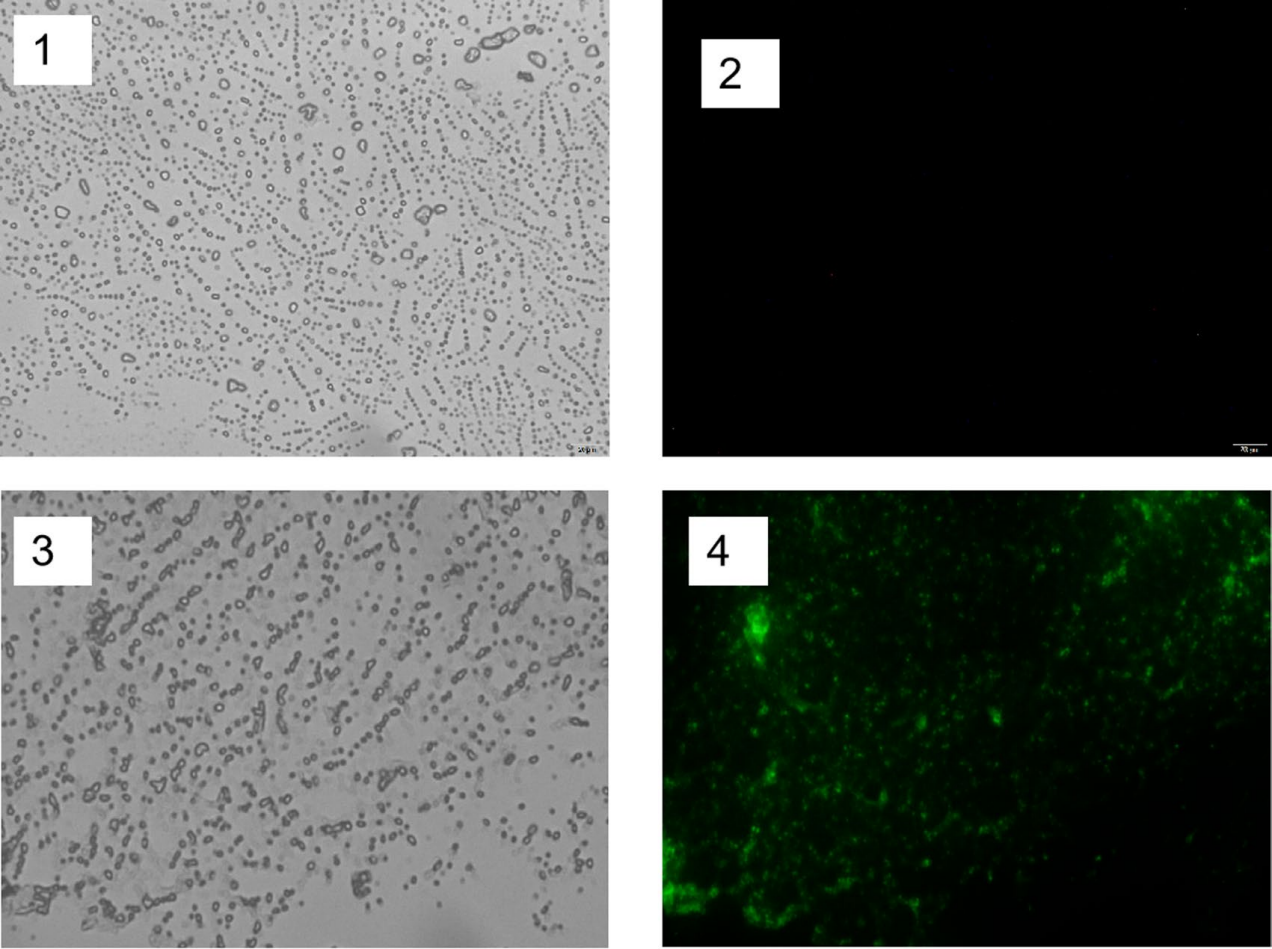
Microscopic Images of Spores with pHS-cotB and pHS-cotB-*mnp* Constructs. **1**: White light image of pHS-cotB spores, displaying the spores under standard illumination. **2**: Fluorescence image of pHS-cotB spores under UV light, expected to show no fluorescence. **3**: White light image of pHS-cotB-*mnp* spores, providing a baseline view of the modified spores. **4**: Fluorescence image of pHS-cotB-*mnp* spores under UV light, highlighting the presence of fluorescence due to the MnP protein

The fusion protein derived from the immobilized MnP spore surface weighed in at 85 kilodaltons (kDa), with the use of recombinant spores harboring cotB plasmids serving as a comparative control. The enzyme activity of CotB-MnP was 62.72U/mL in spores formed by plasmid cotB without target gene. This was validated through SDS-PAGE, as depicted in Fig. 3(a). Further confirmation was provided by Western blot analysis with rabbit serum antibodies, which revealed a distinct band of the anticipated 85 kDa, as illustrated in the WB results in Fig. 3(b). Moreover, the MnP spores underwent incubation with fluorescent antibodies. Figure 4 demonstrate green fluorescence on the surface of the engineered spores CotB-MnP, while the control spores exhibited an absence of fluorescence. Collectively, these findings substantiate the successful expression of MnP on the spore surface via the cotB display system.

### Enzymatic assessment and characterization of MnP

**Fig. 5:**
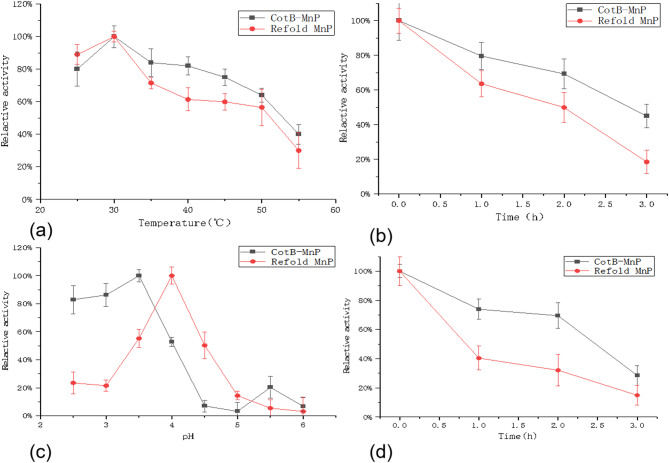
(**a**): The optimal temperature value of CotB-MnP and refolded-MnP; (**b**): Temperature stability of CotB-MnP and refolded-MnP (**c**):The optimum pH of CotB-MnP and refolded-MnP at respective optimum temperature; (**d**): pH tolerance of CotB-MnP and refolded-MnP at respective optimum temperature. Error bars represent SD (*n* = 3)

To determine the optimal temperature for MnP, experiments were conducted within a temperature range of 25℃ to 55℃. As illustrated in Fig. 5(a), both the free *E. coli* refolded-MnP and the CotB-MnP exhibited the highest enzymatic activity at 30℃. However, within the temperature range of 30℃ to 55℃, the relative enzymatic activity of the CotB-MnP was higher than that of the free *E. coli* refolded-MnP, indicating that immobilization technology improves the stability of MnP at high temperatures. Furthermore, to compare the temperature tolerance of the two MnP forms, the residual enzymatic activity was evaluated after incubation at 30℃. As shown in Fig. 5(b), the immobilized enzyme CotB-MnP retained 73.9% of its initial activity after incubation at 30℃ for 1 h incubation, and its activity declined relatively slowly at other temperatures. This further demonstrates the positive effect of immobilization technology on improving the temperature stability of MnP.

The optimal pH conditions for MnP were explored within a pH range of 2.5 to 6.0 at 25℃. The free *E. coli* refolded-MnP exhibited the highest enzymatic activity at pH 4.0, while the immobilized MnP spores achieved optimal activity at pH 3.5(Fig. 5(c)). Further analysis of the data in Fig. 5(d) reveals that after incubation at 30℃ for 1 h, the CotB-MnP retained 79.6% of its initial enzymatic activity, whereas the free *E. coli* refolded-MnP retained only 63.6%. With increasing pH tolerance duration, the CotB-MnP maintained its enzymatic activity better than the free enzyme. These results highlight the advantages of immobilized spores in terms of acidic pH tolerance and enzymatic activity retention.

**Fig. 6:**
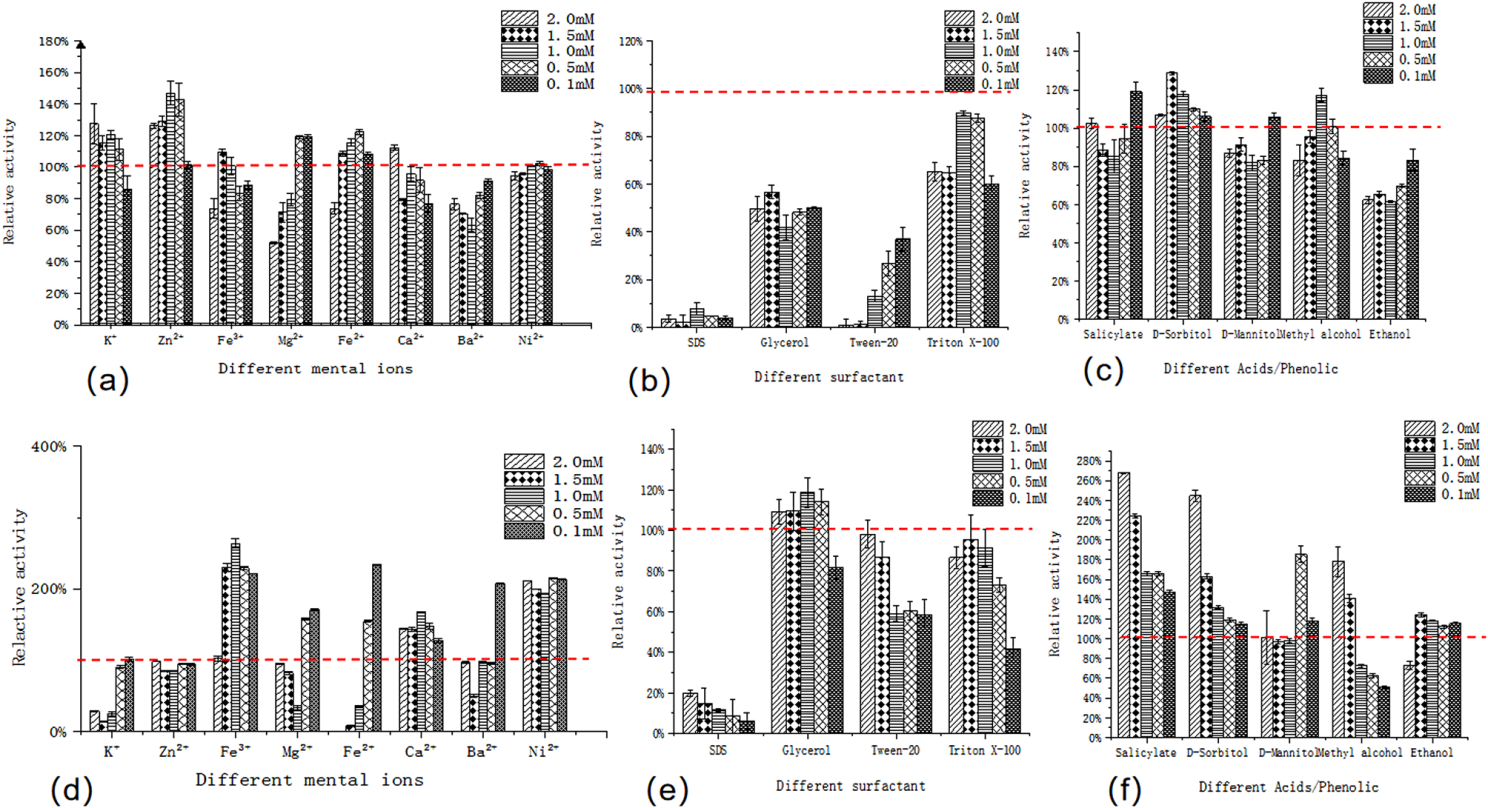
Enzymatic properties of CotB-MnP (panels (**a**)–(**c**)) and refolded-MnP (panels (**d**)–(**f**)). (**a**) The effects of different types and concentrations of metal ions on CotB-MnP; (**b**) The effects of different types and concentrations of surfactants on CotB-MnP; (**c**) The effects of different types and concentrations of acid phenols on CotB-MnP; (**d**) The effects of different types and concentrations of metal ions on refolded-MnP; (**e**) The effects of different types and concentrations of surfactants on refolded-MnP; (**f**) The effects of different types and concentrations of acid phenols on refolded-MnP. The relative activity depicted in each graph represents the peak performance, normalized to 100%, as determined by individual experimental trials. Error bars represent SD (*n* = 3)

Referring to Fig. 6(a) The effects of different types and concentrations of metal ions on CotB-MnP&Fig. 6 (d) The effects of different types and concentrations of metal ions on refolded-MnP, It can be observed that among the eight metal ions tested, different concentrations of Fe^3+^, Mn^2+^, and Ni^2+^ promote refolded-MnP to varying degrees and in a very pronounced manner, whereas CotB-MnP showed remarkable stability in the presence of different metal ions. It is noteworthy that higher concentrations of K^+^ and Zn^2+^ ions promoted the enzymatic activity of cotB-MnP and inhibited that of refolded protein. Lower concentrations of Fe^2+^ and Mg^2+^ ions promote the enzymatic activity of CotB-MnP. It may be that MnP, after displaying on the surface of CotB by the anchoring protein CotB, changes the structure of the protein, so that the metal ions result on the MnP of both structures. In addition, Ni^2+^, Fe^3+^, Ca^2+^ and lower concentrations of Fe^2+^ promoted the enzyme activity of complexed MnP significantly.

Under the influence of the same surfactants, refolded-MnP showed superior activity compared to CotB-MnP. Glycerol slightly promoted the enzymatic activity of refolded MnP. However, surfactants like SDS, Tween-20, and Triton X-100 inhibited its enzymatic activity, with stronger inhibition at lower concentrations(Fig. 6(b)&(e)).

Under the influence of different acids, phenols, and alcohols, the activity of refolded MnP was significantly enhanced by salicylic acid, with higher concentrations showing better promoting effects. On the other hand, the activity of CotB-MnP remained relatively stable under various concentrations of acids and phenols, with D-sorbitol showing a slight promoting effect(Fig. 6(c)&(f).

### Evaluation of spores’ recovery yield post-immobilization of MnP

**Fig. 7:**
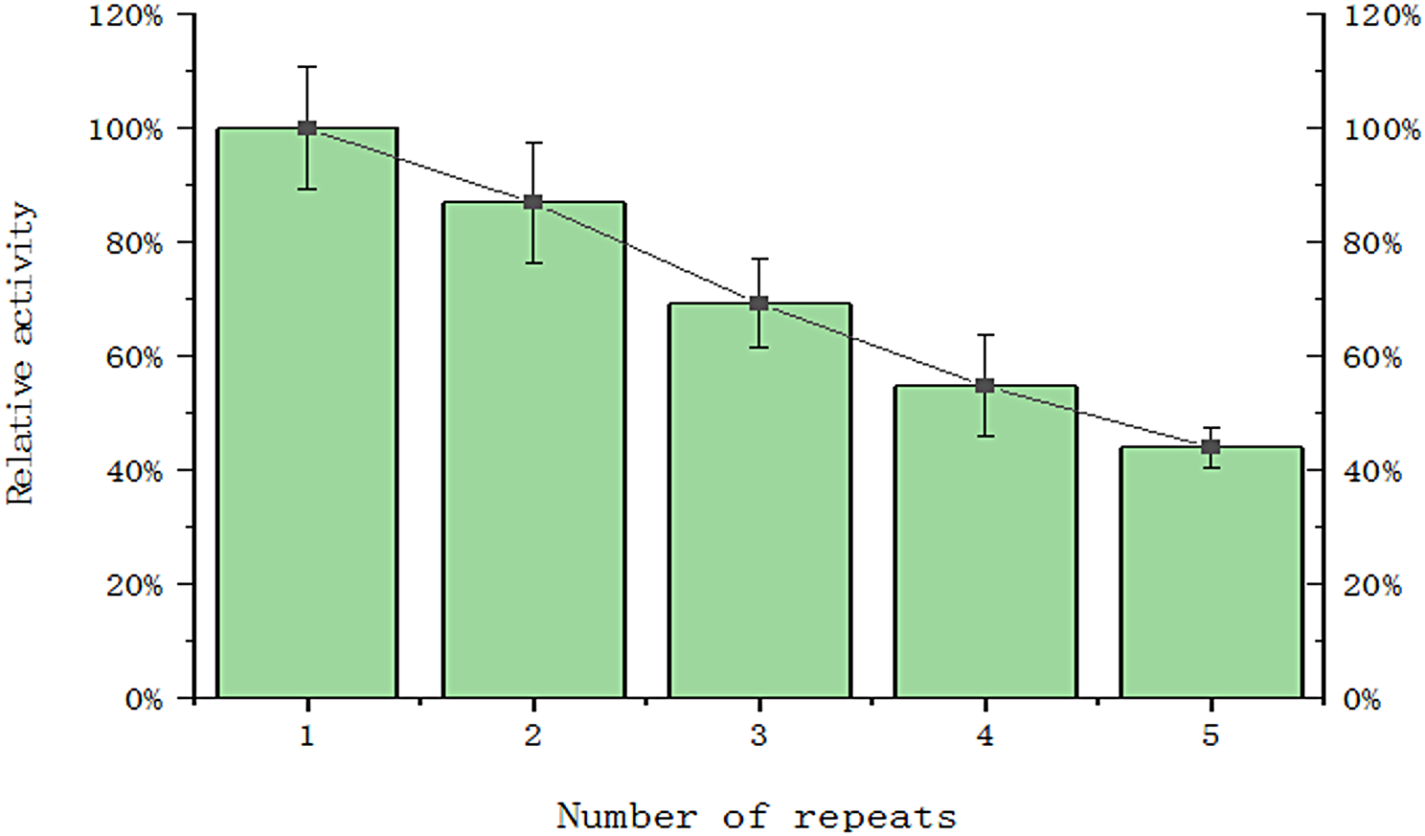
MnP recycling rate after surface display. Error bars represent SD (*n* = 3)

In this study, we evaluated the consecutive recycling enzyme activity of the immobilized CotB-MnP fusion protein to explore its stability and reusability in industrial applications. As shown in Fig. 7, the immobilized CotB-MnP maintained approximately 69% of its original activity over the first three recycling cycles, a result consistent with previous research indicating that the activity of immobilized enzymes is typically retained within the range of 60–70% of the initial value. The preservation of this relative enzyme activity suggests that immobilization technology can effectively protect the structural integrity of CotB-MnP, thereby maintaining its catalytic function during repeated use.

## Discussion

In this investigation, we achieved the isolation of biologically active MnP complexes by employing a series of purification techniques on proteins synthesized within *E. coli*. Following Ni-NTA affinity chromatography, the target protein was eluted from100mM to 500 mM imidazole, yielding a single band at 43 kDa (Fig. 2(c)), consistent with the expected molecular weight of MnP (43 kDa). Subsequently, we engineered a spore surface display system for *Bacillus subtilis*, enabling the targeted immobilization of MnP proteins on the spore surface using the coat protein CotB. This was achieved via the anchoring protein CotB and a linker peptide, allowing for facile isolation of MnP through simple centrifugal washing, which circumvents the laborious steps of protein purification and the energy-intensive freeze-drying techniques required to maintain low temperature and vacuum conditions.

Given the recent advancements in molecular biology technology, *Bacillus subtilis* emerges as an efficient expression system for rapid production of exogenous enzymes(Cui et al. 2018), thereby eliminating the necessity for modifications to the original strain, domestication procedures, and extensive toxicological testing. The *Bacillus subtilis* spore display system not only shortens the production cycle of exogenous enzymes. but also reduces energy consumption while enhancing enzyme stability(Hwang et al. 2013). After MnP was displayed on the surface of subtilis spores through the anchor protein CotB, the enzyme activity reached 62.74 U/mL. Because the MnP displayed by *Bacillus subtilis* could not be quantified, the enzyme activity was higher than that of the recombinant enzyme produced by *E. coli*. Spore-displayed MnP has the potential for direct application in the feedstock fermentation industry, where it can be utilized alongside traditional feed fermenting enzymes such as cellulases and glucose oxidases. This could potentially extend the range of applications for MnP(Chen et al. 2023).

Our analysis comparing the enzymatic characteristics of free refolded MnP and immobilized CotB-MnP showed that the latter exhibits enhanced pH stability at a lower pH of 4.0, indicating a greater capacity to withstand acidic conditions. This advancement paves the way for the utilization of MnP in straw feed fermentation. During feed pretreatment, the pH gradually decreases and remains acidic due to microbial metabolic activity(Zhang et al. 2018). Consequently, enzymes employed in straw pretreatment must possess robust acid resistance to facilitate comprehensive lignin degradation. Furthermore, in contrast to free MnP derived from *Irpex lacteus*, the MnP displayed on the immobilized spore surface exhibits less sensitivity to metal ions, acids, phenols, alcohols, and surfactants present in the test substrates, generally maintaining over 50% of its enzymatic activity. This underscores the improved stability and acid resistance of MnP displayed on the spore surface, making it more suitable for the complex environment of feed fermentation compared to free recombinant MnP.

In parallel, other studies have also demonstrated the merits of spore surface display. Wang Fuli’s research successfully displayed halogenated dehalogenase DhaA on the spore surface via fusion with the CotG protein, enhancing its tolerance to temperature and pH fluctuations (Wang et al. 2019). Similarly, Mingmongkolchai Sirima utilized the *Bacillus subtilis* spore outer shell protein CotG to immobilize *E. coli* phytase (APPA), significantly improving its temperature and pH tolerance, as well as its resistance to pepsin (Mingmongkolchai and Panbangred 2019).

It is well-known that SDS possesses the capability to disrupt protein structure, resulting in denaturation and subsequent loss of activity, potentially explaining its inhibitory effect on MnP activity in both forms. Notably, Tween-20 and Triton X-100 also exhibit inhibitory effects on MnP enzymatic activity, with Tween-20 demonstrating a pronounced inhibitory effect on CotB-MnP, where the inhibitory strength intensifies with increasing concentration. In a related study by Akshita Mehta, similar inhibitory trends were observed for purified lipases in the presence of Tween-20 and Triton X-100(Mehta et al. 2018). This can be attributed to the fact that, as non-ionic detergents, these surfactants may disrupt lipid-lipid and lipid-protein interactions, altering the enzyme’s active site and reducing its substrate binding affinity (Castillo-Sánchez et al. 2021).

It is well-known that SDS possesses the capability to disrupt protein structure, resulting in denaturation and subsequent loss of activity, potentially explaining its inhibitory effect on MnP activity in both forms. Notably, Tween-20 and Triton X-100 also exhibit inhibitory effects on MnP enzymatic activity, with Tween-20 demonstrating a pronounced inhibitory effect on CotB-MnP, where the inhibitory strength intensifies with increasing concentration. In a related study by Akshita Mehta, similar inhibitory trends were observed for purified lipases in the presence of Tween-20 and Triton X-100 (Mehta et al. 2018). This can be attributed to the fact that, as non-ionic detergents, these surfactants may disrupt lipid-lipid and lipid-protein interactions, altering the enzyme’s active site and reducing its substrate binding affinity (Castillo-Sánchez et al. 2021).

In addition, refolded MnP was very sensitive to metal ions, while the immobilized enzyme showed good stability to the effects of different kinds of metal ions at different concentrations. Among them, Ba^2+^ and Ni^2+^ had a slight inhibitory effect on CotB-MnP, and other metal ions such as Fe^2+^, Mg^2+^, Fe^3+^, and Ca^2+^ promoted CotB-MnP enzyme activity at some concentrations, suggesting that the metal ions had a lesser effect on the immobilized MnP. It is worth noting that K^+^ and Zn^2+^ had a promotional effect on CotB-MnP and inhibited the free state of the enzyme activity of refolded MnP. Metal ions may influence enzyme conformation or interact with the spore surface, thereby affecting activity, and their concentration and binding mode directly affect the conformational stability and catalytic efficiency of the enzyme. The reduced inhibition by Zn^2+^ may result from steric hindrance by the spore coat, which limits metal ion access to the enzyme active site—a phenomenon observed in other immobilized systems (Zhang and Yang 2021). Similarly, K^+^-induced inhibition in free MnP could relate to ionic strength effects on enzyme conformation, mitigated by spore surface charge modulation (Wang et al. 2022). In this study, the enzyme activity decreased when the concentration of Zn^2+^ exceeded 2 mM, which may be related to Mg^2+^ site competition or protein aggregation induced by oxidative stress. Potassium salts (e.g., potassium carbonate and potassium phosphate) are sometimes used as food additives to improve the texture and flavor of food products (Du et al. 2020), and potassium ions may also affect pH and electrolyte balance during fermentation (Shi et al. 2023) which in turn affects microbial activity and metabolic pathways. In addition, zinc acts as a coenzyme and activator of enzymes (Hosaka et al. 2009). Zinc ions play a key role in agriculture, food, and feed industries and are important for increasing crop yields, improving the nutritional value of food products, and promoting the healthy growth of animals (Jomova et al. 2022).Therefore, the enzyme activity promoting effect of K^+^ and Zn^2+^ on CotB-MnP is beneficial for future straw feed utilization.

By restricting the molecular structure of MnP, spore display technique may help stabilize the microenvironment of the enzyme’s active site and reduce the interference of water molecules or other molecules in the active site, which further affects the binding of metal ions and acid ions to MnP and makes the immobilized enzyme more stable and acid-resistant (Jia et al. 2014). Acid-tolerant MnP maintains its activity under a variety of environmental conditions, including in fermentation environments with lower pH values, which enables its application in diverse biofeed production processes. The high efficiency and stability of acid-tolerant MnP imply that it can accomplish the pretreatment of straw in a relatively short period, thereby enhancing production efficiency.

Furthermore, it is noteworthy that the same *mnp* gene can yield distinct protein structures when expressed through different expression systems. For instance, the protein derived from the *Streptococcus lactis* F17 gene exhibits a different conformation compared to the soluble expressed protein in the supernatant of *E. coli* cultures. Specifically, the renatured protein adopts a monomer structure, whereas the soluble protein forms an oligomer (Wang et al. 2016). This variation in protein structure was further validated by investigating the enzymatic properties of these two MnP proteins in diverse reaction environments.

On the other hand, MnP has been successfully fixed on various materials such as gelatin, loofah sponge (Li et al. 2010), gel (Cheng et al. 2007), and polyacrylamide gel (Bilal et al. 2016), thereby improving stability. However, the complex fixation process involving acetone may bring potential toxicity and stimulus problems, as well as high costs related to the purification and preparation of enzyme preparations (Bilal et al. 2017). Therefore, this method is considered not applicable to the preprocessing of feed straw.

The cycle utilization efficiency of enzymes is one of the key factors for their successful commercial application. For example, through a simple washing process, spores can maintain their activity, which is an attractive feature in industrial applications. In one study, after three cycles of MnP, its remaining activity remained above 69%, which shows a higher cycle utilization rate of fibrotonase fixed on nylon sponges (Thampraphaphon et al. 2022). This discovery suggests the possibility of commercialization of large-scale production as a potential immobilized enzyme. The surface display technology of *Bacillus subtilis* spores is a promising method for the immobilization and heterogeneous expression of industrial enzymes. However, this technology has some limitations when applying MnP. To achieve effective display, the genetic and metabolic functions need to be finely adjusted. Additionally, to ensure long-term storage and use, the protein on the surface of the spore requires high stability. The transition from laboratory to industrial scale may bring challenges because the stability of protein display may be affected. To overcome these limitations, researchers are exploring various strategies, including genetic engineering, protein engineering, and fermentation process optimization, to enhance the surface display system of *Bacillus subtilis* spores. These efforts aim to improve the expression level, stability, and application potential of the protein. Through continuous research and technological innovation, the surface display system of *Bacillus subtilis* spores is expected to play a more important role in industrial production. The current study focused on acid resistance and thermal stability, future work should address long-term storage stability and scalability in industrial settings.

## Electronic supplementary material

Below is the link to the electronic supplementary material.

Supplementary Material 1

## Data Availability

Data will be made available on request.
