# Supplementary material for: Bacillus subtilis spore surface display enhances manganese peroxidase stability and stress resistance
Source: Bioresour Bioprocess. 2025 Jun 10;12(1):57. doi: 10.1186/s40643-025-00901-9 (PMC12149065; doi:10.1186/s40643-025-00901-9)

#### Supplementary material

Figure S1: Protein quantitation of free fold MnP after purified


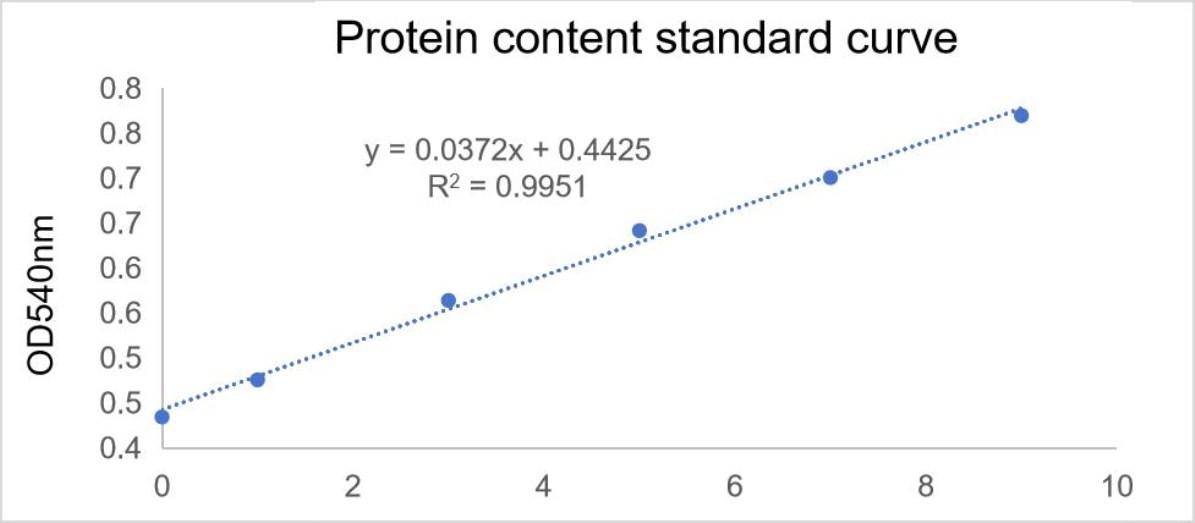


Figure S2: The synthesized MnP gene was used as a template to amplify the MnP target fragment


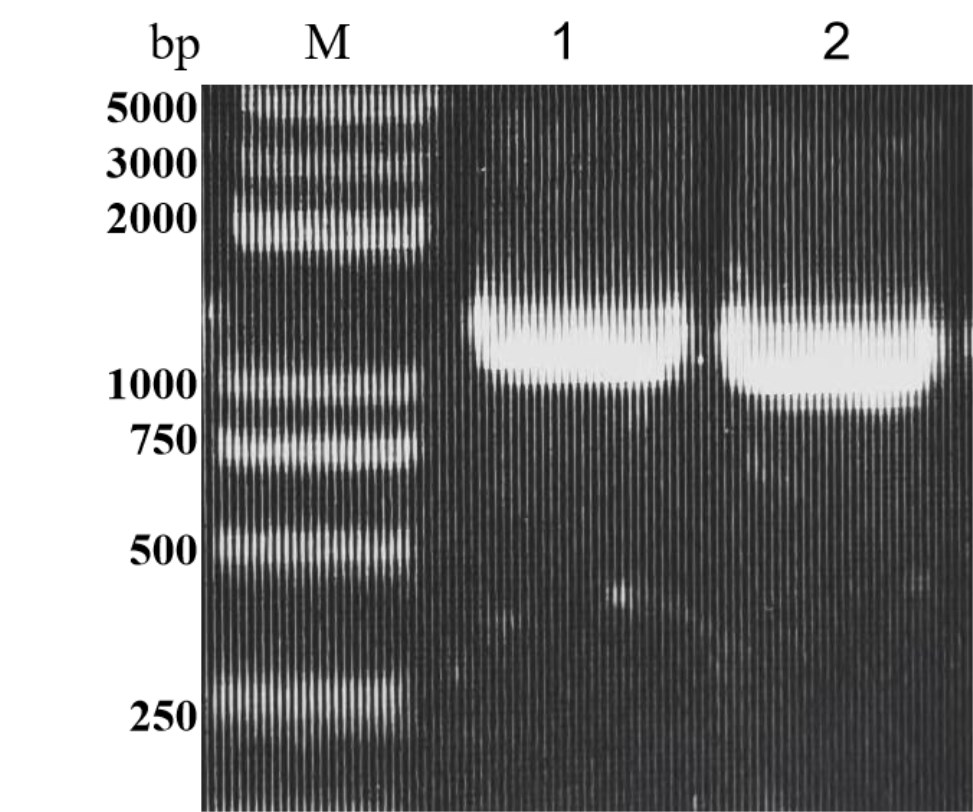


Figure S3: The linearized plasmid


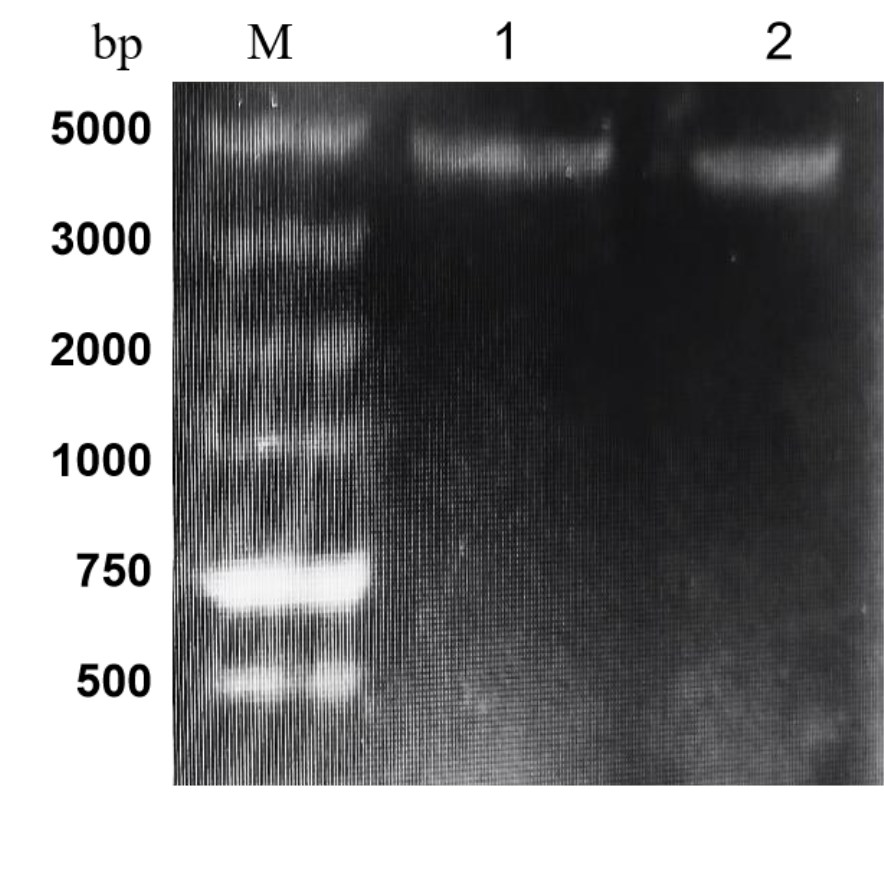

Supplement: Supplementary file 1 — Supplementary Material 1 [file 40643_2025_901_MOESM1_ESM.docx]
